# Vision-based vs. IMU-based upper-limb pose estimation in assisted dressing: a comparative study of positional accuracy and kinematic fidelity

**DOI:** 10.3389/frobt.2026.1844439

**Published:** 2026-07-01

**Authors:** Yasmin Rafiq, Shenglin Wang, Mohammed Al-Nuaimi, Lyudmila Mihaylova, Robert M. Hierons, Sanja Dogramadzi

**Affiliations:** 1 Department Computer Science, The University of Manchester, Manchester, United Kingdom; 2 School of Electrical and Electronic Engineering, The University of Sheffield, Sheffield, United Kingdom; 3 Department of Mechatronics Engineering, College of Engineering, The University of Mosul, Mosul, Iraq; 4 School of Computer Science, The University of Sheffield, Sheffield, United Kingdom

**Keywords:** assisted dressing, convolutional neural networks (CNN), human motion capture, inertial measurement units (IMU), inverse kinematics, occlusion handling, pose estimation, upper-limb kinematics

## Abstract

Accurate estimation of upper-limb kinematics is essential for applications such as rehabilitation assessment and assistive robotics, yet remains challenging in real-world scenarios involving occlusion and physical human interaction. While vision-based pose estimation methods have advanced significantly, their ability to recover reliable joint kinematics under such conditions remains unclear. This paper presents a systematic comparison of vision-based and wearable sensing approaches for upper-limb pose estimation during assisted dressing tasks. A monocular RGB-based convolutional neural network (CNN) and a temporally smoothed variant (CNN_temporal) are evaluated alongside a wearable IMU-based reconstruction method. All approaches are compared against an inverse kinematics (IK) reference derived from VICON motion capture data using participant-specific kinematic models. Performance is assessed using both positional error, measured via global and shoulder-centred mean per-joint position error (MPJPE), and kinematic agreement, measured via elbow flexion/extension angle error. Experiments on a real-world dataset of assisted dressing trials, involving an occupational therapist and three participants, demonstrate that IMU-based estimation provides consistently accurate and stable joint-angle reconstruction (e.g., ∼12° mean absolute error). In contrast, vision-based methods achieve reasonable positional accuracy (MPJPE ∼ 0.20 m) but exhibit substantially larger errors in joint-angle estimation (often exceeding 80 ° ), particularly under occlusion. Temporal smoothing improves positional consistency but does not preserve kinematic fidelity. These results highlight a fundamental limitation of current vision-based approaches for tasks requiring accurate joint kinematics. The findings suggest that integrating inertial sensing or incorporating biomechanical constraints may be necessary to achieve reliable pose estimation in real-world assistive scenarios.

## 1 Introduction

Reliable estimation of human upper-limb motion is a fundamental requirement for safe physical human–robot interaction (pHRI), particularly in assistive applications where robots operate in close proximity to users (Haddadin and Croft, 2016; Goodrich and Schultz, 2008). Tasks such as rehabilitation support and robot-assisted dressing require continuous and accurate estimation of human motion to enable safe adaptation to user behaviour. In these settings, accurate reconstruction of joint kinematics is critical, as control decisions and clinical interpretation depend on meaningful joint-angle information rather than coarse spatial tracking alone.

Vision-based pose estimation methods, particularly those based on convolutional neural networks (CNNs), have seen rapid progress in recent years. Models such as HRNet (Wang et al., 2020) and SimpleBaseline3D (Martinez et al., 2017) enable monocular estimation of 3D human pose and offer a scalable, non-intrusive sensing solution. However, these approaches are typically evaluated on curated datasets and operate on a frame-by-frame basis. Their performance can degrade in real-world assistive scenarios characterised by occlusions, self-contact, and interaction with other people or objects, where visual information is incomplete or ambiguous and temporal inconsistencies can lead to unreliable joint-angle estimates. Occlusion-related challenges during robot-assisted dressing have previously been observed in vision-based tracking systems, particularly when garment interaction causes partial or complete loss of upper-limb visibility (Chance et al., 2018). Recent studies evaluating markerless vision-based pose estimation for upper-limb kinematic analysis have similarly reported limitations in joint-angle accuracy and robustness under occlusion, particularly when compared against reference motion capture systems (Lefebvre et al., 2025; Lam et al., 2025; Francia et al., 2026).

In contrast, wearable measurement units (IMUs) provide direct measurements of segment orientation and are widely used for human motion capture in both laboratory and wearable settings (Roetenberg et al., 2009; Von Marcard et al., 2017; Huang et al., 2018). IMU-based approaches are inherently less sensitive to visual occlusion and can therefore offer more stable estimates of joint kinematics, although they require careful sensor placement and calibration. Despite these complementary strengths, there remains limited understanding of how vision-based and IMU-based methods compare in realistic interaction scenarios, particularly with respect to both positional accuracy and kinematic fidelity. In particular, few works directly compare these approaches under real-world interaction conditions involving occlusion and physical interaction.

Most existing evaluations of vision-based pose estimation methods focus primarily on positional error, typically measured using mean per joint position error (MPJPE) (Martinez et al., 2017; Pavllo et al., 2019; Cai et al., 2019). This reflects the fact that many models are trained using positional supervision, which does not explicitly enforce kinematic consistency. While useful, such metrics do not fully capture the quality of reconstructed motion, as accurate joint positions do not necessarily imply accurate joint angles, particularly in tasks involving complex articulation and interaction.

In this work, we address this gap by systematically comparing vision-based and IMU-based approaches for upper-limb pose estimation using a real-world dataset of assisted dressing trials. The dataset captures interactions between participants and an occupational therapist under both self-dressing (T0) and assisted dressing (T1, T2) conditions, thereby introducing realistic sources of occlusion and variability. An inverse kinematics (IK) model, constructed from VICON motion capture data using participant-specific kinematic models, is used as a reference for evaluating both positional and kinematic accuracy.

We evaluate a frame-wise CNN and a temporally smoothed variant (CNN_temporal) alongside an IMU-based reconstruction method. Performance is assessed using both positional metrics (global and shoulder-centred MPJPE) and elbow flexion/extension angle error, enabling a direct comparison between spatial accuracy and kinematic fidelity across sensing modalities.

The contributions of this paper are as follows.

- A systematic comparison of monocular vision-based and IMU-based upper-limb pose estimation in a real-world assisted dressing scenario.
- An evaluation framework that jointly considers positional accuracy and joint-angle fidelity using an IK-based reference derived from motion capture data.
- An analysis of the limitations of vision-based approaches under occlusion and interaction, and the effect of temporal smoothing on kinematic consistency.

To our knowledge, this study is among the first to systematically compare vision-based and IMU-based upper-limb pose estimation methods in a real-world assisted dressing scenario while jointly evaluating accuracy and kinematic fidelity under occlusion and physical interaction.

The remainder of the paper is organised as follows. Section 2 reviews related work. Section 3 describes the assisted dressing dataset. Section 4 presents the proposed framework. Section 5 details the trial extraction, synchronisation, calibration, and cross-modal alignment procedures. Section 6 presents the evaluation results and analysis. Section 7 discusses the findings, including limitations and directions for future work. Finally, Section 8 concludes the paper.

## 2 Related work

### 2.1 Robot-assisted dressing

Robot-assisted dressing has received increasing attention due to the growing demand for assistive technologies in ageing populations and care environments. Ensuring safe physical human–robot interaction (pHRI) remains a central challenge, particularly in tasks involving close contact and dynamic human motion.

Early work has focused on modelling physical interaction through haptic sensing. For example, Erickson et al. (2018) proposed a deep recurrent model to estimate garment forces from end-effector observations, while Clegg et al. (2020) combined haptic feedback with reinforcement learning to learn dressing policies in simulation. These approaches emphasise force estimation and control, but typically rely on specialised sensing and controlled environments.

Vision-based approaches provide a complementary alternative due to their low cost and ease of deployment. Prior work has used RGB or RGB-D sensing to infer contact and interaction states (Magrini et al., 2014; Ehsani et al., 2020; Koganti et al., 2017). Pignat and Calinon, (2017) employed a hidden semi-Markov model to jointly model human motion and robot actions, using external markers to track hand trajectories during demonstrations. In addition to vision-based perception, some assistive dressing systems incorporate haptic or force-based sensing to detect physical interaction and ensure safe manipulation. While these approaches provide valuable information about contact dynamics, they do not directly address the problem of accurate human motion reconstruction, and are therefore complementary to the pose estimation methods considered in this work.

Despite these advances, reliable perception in real-world dressing scenarios remains challenging due to occlusions, garment interaction, and spontaneous human motion. This motivates the need for robust pose estimation methods that can operate under realistic interaction conditions without reliance on specialised instrumentation.

### 2.2 Vision-based human pose estimation

3D human pose estimation from monocular RGB input is commonly formulated as a regression problem, where the goal is to recover 3D joint positions from 2D observations. Early approaches directly regressed volumetric representations, such as voxel-based likelihoods (Pavlakos et al., 2017) or volumetric heatmaps (Luvizon et al., 2018), achieving improved spatial reasoning at the cost of increased computational complexity.

More recent approaches adopt a two-stage pipeline, where 2D keypoints are first detected and subsequently lifted to 3D (Martinez et al., 2017; Zheng et al., 2021; Zhao et al., 2021). Graph-based models (Zhao et al., 2019) further exploit skeletal structure to model spatial dependencies. While these methods are computationally efficient, their performance is inherently limited by the accuracy of 2D keypoint detection.

Parametric body models such as SMPL (Bogo et al., 2016) incorporate body shape and joint constraints, enabling richer representations but requiring careful initialisation and higher computational cost. Despite strong performance on benchmark datasets, many vision-based approaches are trained using positional supervision and evaluated primarily using joint position error metrics, which may not fully capture kinematic consistency.

Recent work reported the suitability of markerless pose estimation for upper-limb kinematic analysis. Lefebvre et al. (2025) investigated three-dimensional markerless reconstruction of anatomical landmarks in the shoulder and upper limb, highlighting challenges related to joint visibility and reconstruction accuracy. Lam et al. (2025) evaluated markerless motion capture in stroke survivors and demonstrated its feasibility for clinical upper-limb assessment, while noting reduced accuracy under constrained or impaired movement patterns. More recently, Francia et al. (2026) provided a systematic validation across full and partial ranges of motion, reporting strong temporal agreement with reference systems but reduced accuracy at extreme joint configurations, largely due to occlusion and geometric ambiguity. Studies have also highlighted the importance of accurate elbow flexion estimation and appropriate representation of angular errors in upper-limb kinematic analysis and rehabilitation assessment (Bicchi and Colombo, 2024).

These studies demonstrate the growing potential of markerless approaches for upper-limb kinematics, while also highlighting persistent limitations in joint-angle accuracy and robustness under occlusion. However, most evaluations are conducted in controlled laboratory settings, with limited assessment under real-world interaction scenarios involving physical assistance and complex occlusions. In contrast, this work evaluates markerless pose estimation under real-world assisted dressing conditions, where occlusions, interaction dynamics, and task variability introduce additional challenges.

### 2.3 IMU-based motion capture

In contrast to vision-based methods, inertial measurement units (IMUs) provide direct measurements of segment orientation and are widely used for human motion capture in both laboratory and wearable settings (Roetenberg et al., 2009; Von Marcard et al., 2017; Huang et al., 2018). IMU-based approaches are inherently robust to visual occlusion and can provide temporally consistent estimates of joint kinematics through sensor-to-segment modelling and kinematic reconstruction.

Despite their complementary strengths, there remains limited work directly comparing IMU-based and vision-based pose estimation methods in realistic interaction scenarios, particularly with respect to both positional accuracy and kinematic fidelity. In particular, few works evaluate these approaches under real-world interaction conditions.

### 2.4 Occlusions in human pose estimation

Occlusions remain a significant challenge for vision-based pose estimation, particularly in scenarios involving human–object interaction or close physical contact.

Temporal modelling has been widely explored to improve robustness under occlusion. For example, Gu et al. (2021) proposed a temporal regression network with gated convolution to recover occluded joints, while Cheng et al. (2019) used tracking-based approaches to improve temporal consistency. Other methods incorporate occlusion-aware supervision (Ghafoor and Mahmood, 2022) or explicit occlusion reasoning (Liu et al., 2022) to improve prediction reliability.

Multi-modal approaches have also been proposed. For instance, Singh et al. (2024) combined depth and thermal imaging with generative models to address occlusions in healthcare settings. However, such approaches often require additional sensing modalities or complex training strategies, which may limit their applicability in real-time assistive systems.

In contrast, this work focuses on evaluating the robustness of pretrained vision-based pose models under occlusion and comparing their performance with IMU-based kinematic reconstruction in real-world assisted dressing scenarios. This enables a more comprehensive assessment of both positional accuracy and kinematic fidelity under realistic interaction conditions.

## 3 Assisted dressing trials

### 3.1 Participants

We report results from dressing trials involving a professional occupational therapist (OT) and three healthy volunteers (one female and two males), aged 22–32 years, with heights ranging from 160 to 179 cm and weights from 62 to 96 kg.

Participants were instructed to mimic four upper-body spasticity poses defined by Hefter et al. (2012), which are representative of movement patterns commonly observed in stroke patients (Ivanhoe and Reistetter, 2004). These poses primarily affect the configuration of the shoulder, elbow, forearm, and wrist joints.

All participants provided written informed consent for participation and the use of recorded data (including images and videos) in open-access publications. The study was approved by the University of Sheffield Ethics Committee (Reference: 043,182), and all procedures were conducted in accordance with relevant guidelines and regulations.

A total of 12 dressing trials were conducted, corresponding to each combination of participant and spasticity pose. In each trial, the participant was seated while the OT assisted with donning a garment. The average trial duration was approximately 469 s, with rest periods of around 76 s between trials.

Across all trials, RGB video data were recorded at 30fps, yielding approximately 14,000 frames per trial, while motion capture data were recorded at 100fps, resulting in approximately 46,900 marker trajectory samples per trial.

### 3.2 Motion capture

An eight-camera VICON motion capture system was used to record 3D marker trajectories at 100fps during the dressing process. The dressing procedure began with the participant’s left (simulated impaired) arm, followed by the left shoulder, and finally the right (unimpaired) arm.

Markers were placed on anatomically meaningful landmarks, specifically at the base of the middle finger on both hands, enabling the tracking of hand trajectories throughout the dressing task.

Marker occlusions, which occurred due to garment interaction and self-occlusion during dressing, were addressed using interpolation methods provided by the VICON Nexus system. Following data collection, marker trajectories were filtered using a fourth-order low-pass Butterworth filter with a cutoff frequency of 7 Hz to reduce noise while preserving movement dynamics.

### 3.3 Planned disruptions

To capture realistic and varied motion patterns, each dressing scenario included a planned disruption designed to elicit spontaneous and reactive movements from the participant. These disruptions introduce additional variability and complexity into the dataset, reflecting real-world conditions encountered during assisted dressing.

The disruptions included: (i) a simulated fire alarm $\left(d_{1}\right)$ , (ii) a random phone call to the participant $\left(d_{2}\right)$ , (iii) spontaneous interaction with nearby objects $\left(d_{3}\right)$ , and (iv) an obstruction introduced in the OT’s path $\left(d_{4}\right)$ .

Each disruption condition was systematically paired with a spasticity pose, resulting in consistent pose–disruption combinations across participants. All participants experienced the same set of disruption conditions, ensuring comparability across trials. As a result, each trial corresponds to a specific pose–disruption pairing, repeated across participants.

These perturbations result in non-smooth, non-repetitive motion patterns, as well as increased instances of occlusion and rapid pose changes, thereby providing a challenging benchmark for evaluating pose estimation methods across different sensing modalities.

The collected multi-modal dataset forms the basis for evaluating vision-based and IMU-based pose estimation methods, as described in the following section. This design enables evaluation of both positional accuracy and kinematic fidelity across sensing modalities.

## 4 Proposed framework

This section describes the proposed framework for estimating upper-limb pose from vision-based and IMU-based modalities, and outlines how these estimates are generated and subsequently evaluated against an inverse kinematics reference.

### 4.1 Overview

**FIGURE 1:**
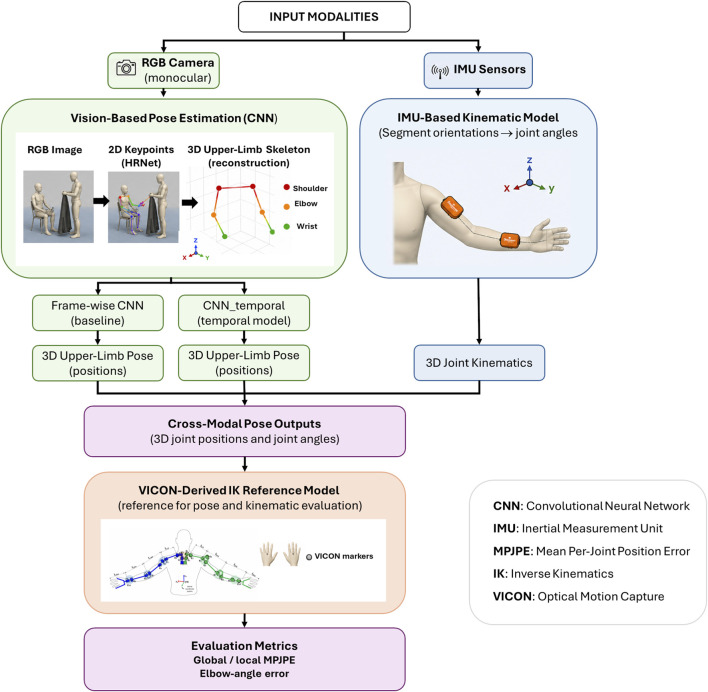
Overview of the proposed framework for comparing vision-based and IMU-based upper-limb pose estimation. Monocular RGB input is processed using a convolutional neural network (CNN) to estimate 3D joint positions, with a temporally smoothed variant (CNN_temporal) applied to the frame-wise predictions. In parallel, IMU measurements are used to reconstruct joint kinematics via a segment-based model, providing joint orientations and angles. All methods are evaluated against a VICON-derived inverse kinematics (IK) reference model using both positional (mean per-joint position error, MPJPE) and kinematic (elbow-angle error) metrics.

The proposed framework enables a structured comparison between vision-based and IMU-based approaches for upper-limb pose estimation under real-world assisted dressing conditions. As illustrated in Figure 1, the system consists of two parallel sensing and reconstruction pipelines: a vision-based pipeline using monocular RGB input, and an IMU-based pipeline using wearable inertial sensors. Both pipelines generate estimates of upper-limb motion, which are evaluated against a common inverse kinematics (IK) reference derived from VICON motion capture data.

The vision-based pipeline follows a top-down pose estimation approach, combining 2D keypoint detection and 3D lifting to produce joint position estimates. Two variants are considered: a frame-wise convolutional neural network (CNN) and a temporally smoothed extension (CNN_temporal), which incorporates sequential information to improve temporal consistency.

In parallel, the IMU-based pipeline reconstructs upper-limb kinematics from segment orientation measurements obtained from multiple sensors attached to the limb. These measurements are used within a segment-based kinematic model to estimate joint angles and corresponding joint positions.

To enable a consistent comparison across modalities, all outputs are evaluated against a VICON-derived IK reference model, which reconstructs participant-specific upper-limb kinematics from marker trajectories. This provides a common representation of both joint positions and joint angles.

The framework is designed to support evaluation using both positional metrics, such as global and shoulder-centred mean per joint position error (MPJPE), and kinematic metrics, specifically elbow-angle error. This allows the relationship between spatial accuracy and kinematic fidelity to be examined directly.

In addition to the core estimation pipelines, the full system includes trial extraction from continuous recordings, cross-modal synchronisation, sensor calibration, and coordinate-frame alignment. These steps are essential for ensuring comparability between modalities and are described separately in the experimental setup and data processing sections.

### 4.2 Vision-based pose estimation

The vision-based pipeline follows a top-down human pose estimation framework (Nguyen and Kresovic, 2022), consisting of three stages: human detection, 2D pose estimation, and 2D-to-3D pose lifting.

In the first stage, a Faster R-CNN detector (Girshick et al., 2014) is used to localise the human subject in each RGB frame and generate bounding boxes. The detected region is then cropped to produce a single-person image, which is passed to the pose estimation network.

For 2D pose estimation, we employ HRNet (Wang et al., 2020), which maintains high-resolution representations throughout the network and aggregates multi-scale features. HRNet predicts K anatomical keypoints corresponding to human joints, represented as heatmaps. For each estimated 2D keypoint, a target heatmap $y_{i}$ is generated using a 2D Gaussian centred at the joint location. The training objective is given by Equation 1.

$$L_{2dKeypoint}=\frac{1}{K}\overset{K}{\underset{i=1}{\sum }}{\left(y_{i}-{\hat{y}}_{i}\right)}^{2},$$ (1)

where ${\hat{y}}_{i}$ denotes the predicted heatmap for keypoint i .

The resulting 2D keypoints are then passed to a 2D-to-3D lifting network based on the SimpleBaseline3D architecture (Martinez et al., 2017). This model takes the 2D joint coordinates as input and predicts the corresponding 3D joint positions, forming a reconstructed upper-limb skeleton.

The output of this pipeline is a set of 3D joint positions for each frame, representing the estimated pose of the upper limb. The CNN model is pre-trained on large-scale human pose datasets and applied in this work without task-specific fine-tuning. As a result, it is optimised to minimise spatial keypoint error rather than to enforce biomechanical constraints or joint-angle consistency.

This distinction is important in the context of this study, where both positional accuracy (MPJPE) and kinematic accuracy (elbow-angle error) are evaluated. The model is not explicitly trained to preserve joint-angle relationships, which has implications for its performance in kinematic reconstruction.

The use of monocular RGB input simplifies deployment but introduces inherent limitations, including depth ambiguity and sensitivity to occlusion. These challenges are particularly relevant in assisted dressing scenarios, where body parts may be partially obscured by the garment or by interaction with an occupational therapist.

### 4.3 Temporal vision-based pose estimation (CNN_temporal)

To improve temporal consistency and robustness under occlusion, a temporally enhanced variant of the vision-based pipeline, denoted CNN_temporal, is introduced. This model extends the frame-wise CNN by incorporating sequential information across multiple frames during the 3D pose estimation stage. The same 2D-to-3D lifting model described in Section 4.2 (SimpleBaseline3D, Martinez et al., 2017) is used here without modification.

In contrast to the frame-wise approach, where each frame is processed independently, CNN_temporal maintains a sliding temporal window of consecutive frames. For each time step, 2D keypoints estimated from the current and previous frames are accumulated into a fixed-length sequence. This sequence is then passed to a temporal 3D pose lifting model, enabling the network to exploit motion continuity when reconstructing 3D joint positions.

In the implemented pipeline, a temporal window of fixed length is maintained using a first-in-first-out buffer, allowing the model to incorporate historical pose information while preserving online processing capability. The temporal lifting stage operates on this sequence of 2D keypoints to generate a temporally smoothed 3D pose estimate for the current frame. The temporal lifting model is based on a sequence-to-sequence architecture operating over fixed-length pose windows.

To improve robustness in the presence of noisy detections and occlusions, additional pre-processing steps are applied prior to temporal lifting. Specifically, low-confidence 2D keypoints are filtered using confidence-based gating, where unreliable detections are replaced with the last reliable observation. Furthermore, optional adaptive filtering is applied to the 2D keypoints to reduce jitter before temporal aggregation.

The resulting CNN_temporal model therefore combines three components: (i) frame-wise 2D keypoint detection, (ii) temporal aggregation of 2D poses, and (iii) sequence-based 3D pose lifting. This design improves the stability of the reconstructed motion, particularly during rapid movement and partial occlusion.

It is important to note that, despite improved temporal smoothness and reduced positional noise, the CNN_temporal model remains trained under a positional objective and does not explicitly enforce biomechanical constraints. As a result, improvements in positional accuracy do not necessarily translate to improved kinematic accuracy, a point examined in the evaluation.

In earlier iterations of this work, additional filtering strategies, including Kalman Filter (KF) and Adaptive Kalman Filter (AKF) variants applied to 2D and 3D keypoints, were explored. However, these approaches did not consistently improve performance and are therefore not included in the final evaluation.

### 4.4 IMU-based kinematic reconstruction

In parallel with the vision-based pipeline, upper-limb motion is reconstructed using wearable inertial measurement units (IMUs). Unlike the vision-based approach, which infers pose from image observations, the IMU-based pipeline directly measures segment motion through inertial sensing, enabling the reconstruction of joint kinematics.

In the experimental setup, four IMUs are attached to the upper limbs, with sensors placed on the forearms and upper arms. Each sensor provides measurements of segment orientation over time. These orientations are represented using unit quaternions q , which provide a compact and singularity-free representation of 3D rotation. For kinematic reconstruction, the quaternion estimates are converted to rotation matrices $R_{i}\in SO\left(3\right)$ , and joint motion is computed from the relative rotation between adjacent limb segments. The estimated orientations form the basis for reconstructing the motion of the corresponding limb segments.

A segment-based kinematic model is employed to map IMU-derived orientations to joint motion. In this model, the upper limb is represented as a chain of rigid segments (upper arm, forearm, and hand), with joints defined between adjacent segments. Joint angles are computed from the relative orientations of connected segments. Given two adjacent segments with orientations $R_{i}$ and $R_{j}$ , where the rotation matrices are obtained from the corresponding quaternions, the relative segment rotation is computed using Equation 2. The relative rotation is defined as:

$$R_{\text{rel}}=R_{i}^{-1}R_{j},$$ (2)

from which joint angles are extracted according to the anatomical joint definitions (e.g., elbow flexion/extension). This formulation enables consistent estimation of joint kinematics directly from segment orientations.

From the reconstructed segment orientations and joint angles, corresponding joint positions are derived using forward kinematics. This provides a representation of the upper-limb pose that is directly comparable to the 3D joint positions estimated by the vision-based pipeline, while also retaining explicit joint-angle information.

The IMU-based approach is inherently well-suited for capturing joint kinematics, as it directly encodes rotational motion. However, accurate reconstruction depends on correct sensor-to-segment mapping and consistent orientation frames. Calibration procedures are therefore required to align IMU measurements with the kinematic model and to ensure consistency with the reference coordinate system.

These calibration and alignment steps, including sensor-to-segment assignment and coordinate-frame harmonisation with the IK reference, are described in the experimental setup and data processing sections.

### 4.5 VICON-derived inverse kinematics reference model

To provide a consistent biomechanical reference for evaluation, upper-limb kinematics are reconstructed from VICON motion capture data using an inverse kinematics (IK) solver. The IK framework maps recorded marker trajectories to joint configurations of a participant-specific kinematic model, enabling the estimation of both joint positions and joint angles. It should be noted that the term “markers” in this section refers specifically to the optical markers tracked by the VICON motion capture system, and is distinct from the IMU sensors used for inertial measurements. The model parameters, including upper-arm and forearm segment lengths, are derived from participant-specific anthropometric measurements based on body height, following standard proportional relationships for upper-limb segments (Lohman et al., 1988). The kinematic chain is rooted at a torso/shoulder reference frame, from which all upper-limb joint positions and orientations are reconstructed, ensuring consistency between model and observed motion.

The kinematic model is defined using the Unified Robot Description Format (URDF), where the upper limb is represented as a chain of rigid segments, including the upper arm, forearm, and hand. These segments are connected through joints that approximate the anatomical structure of the human arm. Simple joints are modelled as single-axis revolute joints, while more complex joints, such as the shoulder, are represented using multiple revolute joints combined with transformation frames to capture multi-axis motion, including abduction/adduction, flexion/extension, and internal/external rotation.

The IK solver uses the Levenberg–Marquardt (LM) optimisation algorithm (Gavin, 2019) to estimate joint configurations that best fit the observed VICON marker trajectories. Unlike conventional motion capture approaches that rely on dense full-body marker sets, the IK reconstruction in this work is driven by a sparse set of markers placed on the dorsal region of the hands near the base of the middle finger, which act as end-effector observations within the optimisation process. The full upper-limb kinematics are recovered by constraining the solution using the participant-specific articulated kinematic model, fixed segment lengths, and anatomically structured joint chains, allowing intermediate joint configurations to be inferred from limited observations. Similar constrained inverse-kinematics approaches for upper-limb musculoskeletal reconstruction from sparse motion-capture observations have previously been investigated in the literature (Fang et al., 2018).

The IK problem is formulated as a non-linear least-squares optimisation, where joint angles are estimated by minimising the error between measured marker positions and those predicted by the forward kinematic chain. The optimisation is therefore constrained by both the observed marker trajectories and the anatomical structure of the participant-specific URDF model, enabling reconstruction of anatomically feasible upper-limb configurations even in the presence of measurement noise or partial marker occlusion.

Participant-specific URDF models are used to account for variations in limb proportions and segment lengths, with marker locations defined relative to the model frames to ensure a consistent mapping between VICON measurements and the kinematic representation. The output of the IK solver consists of joint configurations, from which both joint positions and joint angles are derived, providing a coherent representation of upper-limb motion across all trials.

Although the IK reconstruction is derived from high-precision motion capture data, it remains dependent on the underlying kinematic model, sparse marker configuration, and optimisation assumptions. As such, it is treated as a reference model rather than absolute ground truth, while still providing a consistent and anatomically interpretable basis for cross-modal evaluation.

The pose outputs from each modality are subsequently processed, synchronised, and aligned to a common reference frame to enable direct comparison, as detailed in the following section.

## 5 Trial extraction, synchronisation, calibration and alignment

This section describes the processing pipeline used to prepare and align vision-based and IMU-based pose estimates with the VICON-derived inverse kinematics (IK) reference. The pipeline includes trial extraction from continuous recordings, temporal synchronisation across modalities, IMU calibration, and alignment of all outputs within a common coordinate reference frame to enable fair and consistent comparison.

### 5.1 Trial extraction

The original recordings consist of continuous assisted dressing sequences involving multiple interaction phases. These recordings are segmented into individual trials based on predefined temporal windows corresponding to specific dressing scenarios (e.g., baseline motion, garment-induced occlusion, and assisted interaction).

For each participant and trial, start and end frames are manually defined to isolate relevant motion segments. This ensures that all modalities are evaluated over consistent temporal intervals and under comparable task conditions.

### 5.2 Temporal synchronisation

The different sensing modalities operate at different sampling rates. VICON motion capture data are recorded at a higher frequency and are therefore downsampled to match the frame rate of the RGB video (30 frames per second). This ensures temporal consistency between the IK reference and the vision-based estimates.

IMU data are similarly resampled and aligned to the same temporal resolution. Temporal offsets between modalities are corrected using cross-correlation of corresponding joint trajectories (e.g., wrist position), allowing alignment of signals based on maximal similarity. Following alignment, all modalities are synchronised to ensure that corresponding frames represent the same physical motion. Residual misalignment was observed to be within a small number of frames (typically within one to two frames), which is sufficient for the frame-level comparison performed in this study.

### 5.3 IMU calibration and sensor-to-segment mapping

IMUs were attached to the upper arm and forearm segments to capture segment orientations during the dressing trials. In this study, four wearable Shimmer Wireless Sensor Units were used, each containing a tri-axial accelerometer, gyroscope, and magnetometer to provide 9-DoF motion measurements.

The raw sensor measurements are internally calibrated using device-specific parameters, including offset vectors, sensitivity matrices, and alignment matrices, to convert raw signals into physically meaningful units and to account for sensor-specific biases and coordinate frame inconsistencies. These calibration parameters enable consistent representation of motion across sensors despite differences in their internal coordinate systems.

Segment orientations are represented using unit quaternions, which provide a compact and singularity-free representation of 3D rotation. Orientations are obtained using the onboard sensor fusion provided by the Shimmer platform to produce drift-reduced segment orientations. This approach provides stable orientation estimates over the relatively short duration of the trials considered in this study which are converted to rotation matrices and mapped to the corresponding segments of the upper-limb kinematic model. Joint motion is then computed from the relative rotation between adjacent segments, enabling estimation of joint angles such as elbow flexion/extension.

In addition to the temporal synchronisation described in Section 5.2, a spatial calibration procedure was performed to align each sensor frame with the corresponding anatomical reference frame. Prior to data collection, each sensor frame was calibrated against the corresponding anatomical segment frame. This calibration ensures consistency between the IMU coordinate frames and the kinematic model, allowing accurate reconstruction of joint kinematics from relative segment orientations.

To improve robustness, IMU signals were smoothed to reduce high-frequency noise prior to reconstruction. While IMU-based estimation provides stable orientation measurements, it is subject to potential sources of error, including sensor drift and calibration inaccuracies, particularly over longer durations. In this study, the relatively short trial durations and calibration procedure mitigate these effects, although residual drift may still contribute to minor deviations in the reconstructed joint angles.

### 5.4 Coordinate frame alignment

To enable comparison across modalities, all pose estimates were transformed into a common coordinate frame. The VICON coordinate system was used as the reference frame.

Vision-based 3D pose estimates and IMU-based reconstructions were aligned to this frame through rigid transformations, accounting for differences in origin, orientation, and scale. A shoulder-centred coordinate system was additionally used for relative error evaluation, reducing sensitivity to global position offsets.

### 5.5 Cross-modal alignment and preprocessing

Following temporal and spatial alignment, additional preprocessing steps are applied to ensure consistency across modalities. These include normalising sequence lengths, handling missing or unreliable measurements, and ensuring that all modalities produce outputs for the same set of frames.

For the vision-based pipeline, low-confidence keypoints are identified based on the confidence scores provided by the 2D pose estimator (HRNet). A threshold τ=0.35 is applied, below which detections are considered unreliable. The threshold was selected empirically through preliminary validation experiments, balancing the need to suppress unreliable detections while retaining sufficient valid observations for downstream analysis. Lower thresholds resulted in noisy and unstable joint trajectories, while higher thresholds removed an excessive number of valid detections, particularly during partial occlusion. In practice, a value of τ=0.35 provided a stable trade-off between noise suppression and temporal continuity of the reconstructed trajectories. A small proportion of frames required imputation under this threshold, primarily in regions affected by occlusion. For such keypoints, the corresponding 3D positions are either filtered out or replaced using the last reliable estimate, following the confidence-based gating strategy described in Section 4. This ensures temporal continuity while preventing noisy detections from degrading the reconstructed pose.

For the IMU-based pipeline, temporal smoothing is applied to the reconstructed segment orientations to mitigate sensor noise and minor calibration inconsistencies. This step is lightweight and preserves the underlying motion dynamics.

The IK reference provides consistent joint configurations across all frames.

These steps ensure that all three modalities—vision-based estimation, IMU-based reconstruction, and IK reference—are directly comparable under a unified evaluation framework.

## 6 Evaluation

This section evaluates the accuracy and kinematic consistency of the pose estimation pipelines across vision-based, temporal, and IMU-based approaches. The evaluation is conducted using a common inverse kinematics (IK)-derived reference, enabling direct cross-modal comparison under a unified representation. All modalities are evaluated under identical temporal alignment and coordinate normalisation procedures to ensure a fair comparison.

Performance is assessed using two complementary metrics: mean per joint position error (MPJPE), which quantifies spatial accuracy in 3D space, and elbow-angle deviation, which captures kinematic fidelity. While MPJPE reflects positional agreement with the reference model, joint-angle deviation provides insight into the correctness of the reconstructed motion, particularly for articulated joints.

The IK-derived reconstruction is treated as a consistent biomechanical reference rather than absolute ground truth, allowing meaningful comparison between modalities while acknowledging modelling assumptions. This dual-metric evaluation enables a comprehensive assessment of pose estimation performance, highlighting differences between spatial accuracy and kinematic realism.

### 6.1 Experimental setup

We compared cross-modal agreement between inverse kinematics (IK), an IMU-based reconstruction, a frame-wise convolutional neural network (CNN), and a temporally smoothed CNN variant (CNN_temporal). IK was used as the reference, and the remaining methods were evaluated against it using both positional and angular metrics. The IK solution is reconstructed from VICON marker data and serves as a consistent reference for cross-modal comparison, rather than absolute ground truth.

To ensure clarity in the evaluation, both positional and kinematic errors are reported using explicitly defined metrics. Positional accuracy is quantified using Mean Per Joint Position Error (MPJPE), computed as the Euclidean distance between estimated and reference joint positions. We report both *global MPJPE*, measured in the original coordinate frame, and *local MPJPE*, computed after translating all joints relative to the shoulder centre, thereby isolating limb configuration error from global translation effects. Local MPJPE is evaluated over the elbow and wrist joints of both arms, and therefore reflects relative upper-limb configuration error rather than absolute position.

Joint angle accuracy is evaluated using the elbow flexion/extension angle, defined as the angle formed at the elbow by the shoulder–elbow and elbow–wrist segment vectors. Specifically, the angle is computed from the vectors connecting the shoulder to the elbow and the elbow to the wrist joints. An angle of approximately 180° corresponds to full elbow extension, while smaller angles indicate increasing flexion. Angular errors are reported using the mean absolute error (MAE), in degrees, between the estimated and reference elbow angles, which is commonly used in upper-limb kinematic validation studies (Bicchi and Colombo, 2024).

The elbow joint was selected as a representative articulation because it has well-defined kinematics and can be compared robustly across modalities, without the additional ambiguity associated with multi-degree-of-freedom joints such as the shoulder.

The analysis includes trials from participants P1 and P2 across conditions T0, T1, and T2, together with a separate analysis of participant P7 in trial T0, where the left and right arms were evaluated independently. Trials are denoted using the format P# T#, where P indicates the participant and T the trial index. Summary results are reported in Table 1, with key trends discussed in the following sections. Although the full dataset includes trials T0–T4, the evaluation focuses on T0–T2, which provide sufficient variation in interaction conditions while maintaining consistent data quality across modalities.

**TABLE 1:** Summary of mean absolute positional and angular errors across all analysed trials. Global and local MPJPE are reported in metres as mean Euclidean distance errors, and elbow-angle error is reported in degrees as mean absolute error (MAE). Lower values indicate better agreement with the IK reference.

Trial	Global MPJPE (m)			Local MPJPE (m)			Elbow error (deg)		
	IMU	CNN	CNN_temp	IMU	CNN	CNN_temp	IMU	CNN	CNN_temp
P1 T0	0.072	0.193	0.192	0.301	0.584	0.596	25.0	73.3	94.7
P1 T1	0.072	0.199	0.164	0.210	0.676	0.585	16.9	97.1	105.5
P1 T2	0.072	0.204	0.061	0.243	0.658	0.590	19.2	75.5	101.9
P2 T0	0.072	0.168	0.064	0.257	0.579	0.434	28.2	65.0	80.7
P2 T1	0.072	0.168	0.132	0.188	0.617	0.528	20.1	78.2	94.5
P2 T2	0.072	0.169	0.070	0.217	0.602	0.504	29.2	71.7	85.6
P7 T0 (L)	0.072	0.169	0.043	0.200	0.611	0.552	24.6	28.9	79.6
P7 T0 (R)	0.072	0.198	0.107	0.157	0.641	0.548	4.1	36.6	118.4
Average	**0.072**	**0.184**	**0.104**	**0.222**	**0.621**	**0.542**	**20.9**	**65.7**	**95.1**

Bold values indicate the average error across all analysed trials for each method and evaluation metric.

Although the original dressing dataset included recordings from seven participants, only participants P1, P2 and P7 were retained for the present evaluation due to incomplete or noisy data in several of the remaining trials across sensing modalities.

Trials were conducted under three conditions. In T0, participants performed dressing tasks independently (self-dressing). In T1 and T2, dressing was assisted by an occupational therapist, introducing additional interaction as well as partial occlusion from both the therapist and the garment.

Across all trials, the right arm corresponds to the participant’s unaffected limb, which remained relatively free-moving and less occluded. In contrast, the left arm was more frequently occluded due to both the dressing task and therapist interaction. This asymmetry is important when interpreting differences in model performance between limbs, as accurate joint trajectories do not necessarily imply physically consistent joint configurations.

The results are analysed to compare performance across sensing modalities and evaluation metrics, with particular emphasis on the relationship between positional accuracy and kinematic fidelity. Differences between methods are interpreted in the context of sensing characteristics, occlusion sensitivity, and temporal consistency.

### 6.2 Elbow-angle accuracy

**FIGURE 2:**
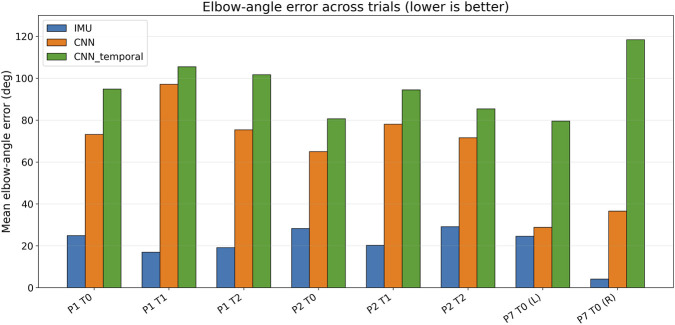
Mean elbow-angle error across all analysed trials for the IMU, CNN, and temporally smoothed CNN models. Lower values indicate closer agreement with the IK reference.

Mean elbow-angle error across trials is shown in Figure 2. The IMU-based estimates consistently produced the lowest angular error relative to the IK reference. Across the standard trials, IMU errors were typically in the range of approximately 17° to 29° , whereas the CNN errors ranged from around 65° to 97° . The temporally smoothed CNN produced the largest errors overall, exceeding 100° in several trials.

The same pattern is visible in the P7 T0 analysis. For the left arm, the IMU error remains substantially lower than both CNN-based approaches, while for the right arm the difference is particularly pronounced, with the temporal model showing a marked increase in error relative to both IMU and CNN.

These results indicate that temporal smoothing does not improve joint-angle agreement. While the CNN_temporal outputs appear smoother, this comes at the cost of increased deviation from the IK reference.

Across all trials, the IMU-based approach consistently achieves the lowest joint-angle error, indicating superior kinematic reconstruction. In contrast, vision-based methods (CNN and CNN_temporal) exhibit larger deviations in elbow angle, despite achieving competitive positional accuracy in terms of MPJPE. This highlights a key discrepancy between spatial and kinematic performance, where accurate joint positions do not necessarily correspond to physically consistent joint configurations. This reinforces that evaluation based solely on positional error may overestimate the practical usefulness of vision-based pose estimation in applications requiring accurate joint kinematics.

Temporal smoothing (CNN_temporal) improves positional stability compared to the frame-wise CNN, as reflected by reduced MPJPE in several trials. However, this improvement does not translate to better kinematic fidelity, with elbow-angle errors remaining comparable or, in some cases, increasing. This suggests that temporal filtering alone is insufficient to enforce biomechanical consistency in vision-based pose estimation.

### 6.3 Positional accuracy

**FIGURE 3:**
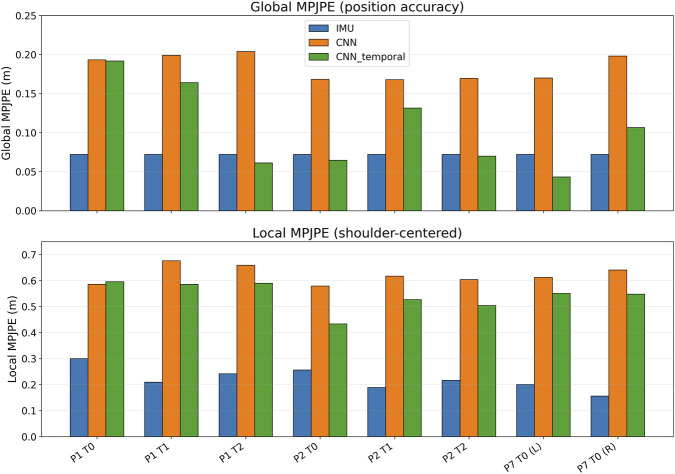
Global and local MPJPE across all analysed trials for the IMU, CNN, and CNN_temporal models.

Global and local MPJPE results are shown in Figure 3. The IMU-based reconstruction again yielded the lowest positional error across all trials. Global MPJPE remained approximately constant at around 0.07 m, while local MPJPE ranged between approximately 0.16 m and 0.30 m.

The CNN produced substantially larger errors, with global MPJPE around 0.17–0.20 m and local MPJPE typically above 0.55 m. Within the vision-based methods, CNN_temporal consistently reduces MPJPE relative to the frame-wise CNN, although it remains less accurate than the IMU-based reconstruction. In most trials, this reduction is more pronounced for the local metric.

These results indicate that while temporal modelling improves positional consistency in the vision-based pipeline, it does not close the gap to the IMU-based approach. Taken together with the angular results, this indicates that improved positional agreement does not necessarily correspond to improved kinematic agreement.

### 6.4 Qualitative temporal behaviour

**FIGURE 4:**
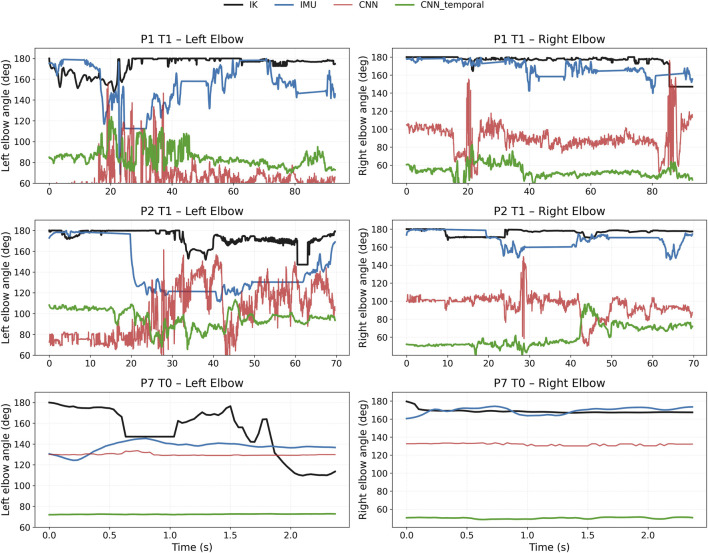
Representative elbow-angle trajectories for selected trials comparing IK, IMU, CNN, and CNN_temporal estimates.

Representative elbow-angle trajectories are shown in Figure 4. Across the selected trials, the IMU-based estimates generally follow the IK reference both in magnitude and overall temporal pattern.

The CNN predictions show larger deviations and increased variability, particularly during more dynamic segments of the motion. The temporally smoothed CNN reduces short-term fluctuations but produces trajectories that are compressed in range and less responsive to changes in the underlying motion.

This behaviour is most evident in the P7 T0 examples, where the CNN_temporal output remains close to a constant value despite visible variation in the IK trajectory. Similar, although less extreme, patterns can also be seen in the P1 and P2 trials.

This further supports the observation that temporal smoothing improves visual stability but does not preserve the underlying joint kinematics. Overall, the qualitative results are consistent with the quantitative analysis: the IMU-based reconstruction provides the closest agreement with IK, while temporal smoothing reduces variability but does not preserve joint-angle behaviour.

Overall, the results demonstrate that IMU-based estimation is more reliable for recovering joint kinematics, while vision-based methods provide reasonable spatial tracking but lack consistency in joint-angle reconstruction, particularly under occlusion and interaction. The improved kinematic performance of the IMU-based approach can be attributed to its direct measurement of segment orientations, enabling consistent reconstruction of joint rotations independent of visual occlusions or missing observations.

## 7 Discussion

This study evaluated the accuracy of IMU- and vision-based approaches for estimating upper-limb kinematics, with a focus on elbow-angle reconstruction and joint position errors. The results reveal a systematic divergence between positional accuracy and kinematic fidelity across methods, with important implications for the design and evaluation of pose estimation systems in real-world assistive applications.

### 7.1 Position accuracy versus kinematic accuracy

A consistent finding across trials is that lower positional error does not necessarily translate into improved joint-angle estimation. While the CNN_temporal model achieved the lowest MPJPE among the vision-based methods in most conditions (Figure 3), it exhibited the largest elbow-angle errors (Figure 2). This decoupling indicates that optimising for spatial keypoint accuracy alone is insufficient for recovering anatomically meaningful joint kinematics.

This behaviour is likely related to the training objective of the CNN models, which are optimised for positional accuracy rather than joint-angle consistency. As a result, improvements in MPJPE do not necessarily reflect improved kinematic reconstruction.

One possible explanation is that temporal smoothing, while beneficial for reducing frame-to-frame positional noise, attenuates high-frequency variations that are critical for accurately resolving joint angles. As a result, trajectories may appear spatially consistent but remain kinematically biased, particularly during dynamic phases of motion. These findings suggest that evaluation based solely on positional metrics such as MPJPE may overestimate the practical reliability of vision-based pose estimation in applications where accurate joint kinematics are required.

These observations are consistent with recent validation studies of markerless upper-limb kinematics. Lefebvre et al. (2025), Lam et al. (2025) report that markerless systems can capture overall motion patterns but exhibit reduced accuracy in joint-angle estimation, particularly under constrained or occluded conditions. Similarly, Francia et al. (2026) demonstrate that while temporal agreement with reference systems is often strong, systematic errors arise at extreme joint configurations due to occlusion and geometric ambiguity.

The results of this study extend these findings to a real-world assisted dressing scenario, where occlusions from garments and therapist interaction further exacerbate these limitations. In particular, the large deviations observed in elbow-angle estimation, despite reasonable positional accuracy, reinforce the need for evaluation frameworks that explicitly consider kinematic fidelity in addition to spatial accuracy, particularly in real-world assistive applications.

### 7.2 Performance of IMU-based estimation

Across all participants and trials, the IMU-based approach consistently yielded the lowest elbow-angle errors. This is expected, as IMUs directly capture segment orientations, providing a more direct measurement of joint kinematics compared to vision-based inference. The stability observed in the time-series plots (Figure 4) further supports the robustness of IMU-derived estimates, particularly during rapid or complex movements.

However, while IMUs perform well in controlled conditions, their reliance on sensor placement and calibration may limit usability in unconstrained environments. Small misalignments or drift can introduce systematic errors, which are not explicitly addressed in this study but remain an important consideration for real-world applications.

This highlights the complementary strengths of the two modalities: IMUs provide accurate and stable kinematic measurements, while vision-based approaches offer greater flexibility and ease of deployment but with reduced kinematic reliability.

### 7.3 Limitations of vision-based methods

The CNN and CNN_temporal approaches both show substantially higher elbow-angle errors compared to IMUs, despite reasonable performance in positional metrics. This discrepancy is particularly evident in trials involving larger ranges of motion or potential occlusions (e.g., P1 T1 and P2 T1), where the angle estimates exhibit increased variability and bias (Figure 4).

The temporal model improves positional consistency but does not resolve underlying geometric ambiguities in joint reconstruction. In some cases, it appears to enforce overly smooth trajectories that diverge from the reference kinematics. This highlights a key limitation of purely data-driven approaches: without explicit biomechanical constraints, accurate reconstruction of joint angles remains challenging.

It is also important to note that the vision-based pipeline relies on a single monocular camera, which limits the available 3D information and increases sensitivity to occlusion and depth ambiguity. Multi-view approaches may help mitigate these issues and improve reconstruction accuracy.

This effect is more pronounced in assisted dressing conditions (T1 and T2), where occlusions introduced by the therapist and garment further complicate visual tracking. Differences between left and right arms are also consistent with this interpretation, with the more occluded limb exhibiting greater variability in angle estimation. These limitations are particularly critical in assistive scenarios, where inaccurate joint-angle estimation may directly affect system safety and interaction quality.

### 7.4 Special case: Independent arm analysis (P7 T0)

The analysis of participant P7, where left and right arms were evaluated independently, provides additional insight into model behaviour. While positional errors for the CNN_temporal model remained relatively low, elbow-angle errors were particularly large, especially for the right arm. The time-series plots indicate limited variation in the predicted angles, suggesting a failure to capture meaningful joint articulation in this scenario.

This case underscores the sensitivity of vision-based models to input conditions and data distribution, particularly when test scenarios deviate from the training data. When the motion pattern deviates from those represented in the training data, the models may default to biased or overly simplified predictions.

### 7.5 Implications for practical applications

The results indicate that IMU-based systems are more reliable for applications requiring accurate joint-angle estimation, such as clinical assessment or biomechanical analysis. Vision-based approaches, particularly those enhanced with temporal modelling, may be better suited for applications where coarse motion tracking or positional consistency is sufficient.

More broadly, these findings highlight the need for hybrid approaches that combine the strengths of both modalities. Integrating IMU data with vision-based models, or incorporating biomechanical constraints into learning frameworks, may help bridge the gap between positional and kinematic accuracy. These findings emphasise the importance of selecting sensing modalities and evaluation metrics based on the specific requirements of the application, particularly when accurate joint kinematics are critical.

### 7.6 Limitations and future work

This study has several limitations that should be considered when interpreting the results. First, the evaluation is based on a small participant cohort, and although multiple trials and interaction conditions are analysed, the findings should be interpreted as indicative rather than statistically generalisable. Future work will extend the evaluation to a larger and more diverse participant population.

Second, the CNN-based pose estimation models are applied without task-specific fine-tuning. While this allows evaluation of their generalisation capability in real-world assisted dressing scenarios, it also introduces a domain gap between the training data and the target task, particularly under conditions of occlusion and human–human interaction. This mismatch may contribute to reduced kinematic fidelity observed in the vision-based approaches.

Third, the IK reconstruction, while derived from high-precision VICON data, is dependent on the underlying kinematic model and the use of a minimal marker set. This may introduce uncertainty in joint centre estimation, particularly for proximal joints, and should be considered when interpreting discrepancies between modalities.

Finally, IMU-based reconstruction is subject to sources of error such as sensor drift, calibration inaccuracies, and sensitivity to initial alignment. Although these effects are mitigated by the relatively short trial durations and calibration procedures used in this study, residual errors may still influence the reconstructed joint trajectories and angles.

Future work should investigate larger datasets, improved calibration strategies, and approaches for enhancing kinematic consistency in vision-based models. In particular, incorporating biomechanical constraints, multi-view geometry, or hybrid sensor fusion approaches may provide a pathway toward more accurate and robust motion reconstruction.

## 8 Conclusion

This study compared IMU- and vision-based approaches for estimating upper-limb kinematics during dressing tasks, with a focus on elbow-angle accuracy and joint position error. The evaluation was conducted on real-world assisted dressing trials, providing a challenging test case involving occlusions, external interactions, and unconstrained motion. A key aspect of this work is the use of an inverse kinematics (IK) reference constructed from a minimal VICON marker set, enabling consistent and anatomically interpretable reconstruction of upper-limb kinematics across all trials.

The results show that IMU-based estimation consistently provides more accurate and stable joint-angle reconstruction, while vision-based methods exhibit substantially larger deviations in kinematic estimates. Within the vision-based approaches, temporal modelling reduces positional error relative to the frame-wise CNN, but does not improve and may in some cases degrade joint-angle accuracy.

A central finding is that improvements in positional accuracy do not necessarily translate into improved joint-angle estimation. While the CNN_temporal model achieved the lowest MPJPE among the vision-based methods in most trials, it exhibited the largest errors in elbow-angle reconstruction. This highlights a fundamental limitation of current vision-based approaches when applied to tasks requiring precise joint kinematics.

Performance differences were also influenced by task conditions, particularly in assisted dressing scenarios where occlusions and external interactions introduce additional complexity. These factors disproportionately affect vision-based methods, especially under a monocular (single-camera) setup where depth ambiguity and occlusion cannot be fully resolved. Multi-view configurations may alleviate some of these limitations, while IMU-based estimates remain comparatively robust.

Overall, the results indicate that IMU-based systems are more suitable for applications requiring accurate joint-angle estimation, such as clinical assessment and rehabilitation monitoring. Vision-based approaches, while flexible and easy to deploy, currently provide more reliable positional estimates than kinematic ones in this context.

Future work should explore hybrid approaches that integrate vision and inertial sensing, as well as methods that incorporate biomechanical constraints or multi-view geometry to improve kinematic consistency in vision-based models. More broadly, achieving reliable pose estimation in real-world assistive scenarios will require approaches that jointly consider spatial accuracy, kinematic fidelity, temporal consistency, and robustness to occlusion.

## Data Availability

The raw data supporting the conclusions of this article will be made available by the authors, without undue reservation.
